# Editorial: The role of physical and biological gut barriers in modulating crosstalk between the microbiota and the immune system

**DOI:** 10.3389/fimmu.2024.1448642

**Published:** 2024-07-19

**Authors:** Vittoria Palmieri, Marika Falcone

**Affiliations:** Autoimmune Pathogenesis Unit, IRCCS San Raffaele Scientific Institute, Milan, Italy

**Keywords:** gut barrier disruption, microbiota, mucosal immunity, autoimmune disease, allergy

The intestine has several means to maintain immune homeostasis and avoid inflammation despite the massive antigenic stimulation from food components and commensal bacteria that are present in the gut mucosa. These include physical barriers such as the mucus layer, the intestinal epithelial barrier (IEB) and the gut vascular barrier (GVB). The integrated response of these combined defense systems is fundamental to containing microbes and their products within the intestine and preventing their systemic spread and ability to activate immune and autoimmune responses in the gut and in extra-intestinal tissues. The main function of the mucus layer is to limit contact between the gut mucosa and harmful molecules present in the intestinal lumen but it is also fundamental in regulating the interaction between the commensal microbiota and the immune system. For example, the mucus layer contains mucins that play key immune regulatory functions (1, 2), such as MUC2, which induces tolerogenic dendritic cells (3). Furthermore, the mucus layer hosts commensal species that are crucial for modulating immunity such as short-chain fatty acid-producing bacteria (4, 5). The IEB is represented by a single layer of epithelial cells held together by a complex junctional system consisting of tight junctions, adherens junctions and desmosomes. Originally, the IEB was believed to be an impermeable barrier that blocks the paracellular passage of macromolecules, but it is now clear that the IEB and the tight junctions are dynamic structures that regulate the continuous antigen trafficking between the intestinal lumen and the gut mucosa (6). Molecules, including bacterial and food antigens, that cross the IEB reach the lymphatic circulation and stimulate immune cells either through pattern recognition receptors on innate immune cell subsets such as DCs or through TCR-mediated antigenic stimulation with molecular mimicry mechanism (7). Finally, the GVB has been recently identified as another important intestinal barrier structure that determines which microbial components can migrate from the gut into the systemic circulation to reach the liver, spleen and peripheral organs and possibly stimulate innate and adaptive immune responses (8).

In this Research Topic several reports have demonstrated the key role of the gut barrier in preventing abnormal immune activation in the gut and in extra-intestinal tissues in sepsis, allergies, and autoimmune diseases like multiple sclerosis (MS) and type 1 diabetes (T1D).

The importance of the gut microbiota in modulating the pathogenesis of autoimmune T1D has been amply demonstrated both in humans and in preclinical models (9, 10). Here, two papers have highlighted the innovative aspects of this interaction. Miranda-Ribera et al. reviewed the lines of evidence supporting a causal link between increased gut permeability and T1D pathogenesis, supporting the notion that uncontrolled antigen trafficking leads to inflammation and skewed effector Th17 cell responses that favor T1D. On the other hand, Pearson et al. demonstrated that enteric viruses of the norovirus family protect against T1D by reducing inflammation and favoring the differentiation of Treg cells in pancreatic lymph nodes. In their review, Jayasimhan and Mariño proposed the innovative concept that intestinal inflammation and breakage of the GB promote T1D pathogenesis by exposing enteric glial cell antigens, which are potential autoantigens in T1D. GB integrity is also important for other extra-intestinal autoimmune diseases. For example, Buscarinu et al. collected in their review numerous reports indicating that intestinal permeability is altered in MS patients, resulting in bacterial translocation and passage of microbiota-derived endotoxins and metabolites into the systemic circulation, which have a pro-inflammatory effect on the central nervous system. GB integrity is also essential in regulating allergic reactions as demonstrated by the work of Ruohtula et al. who showed that the presence of a microbial community dominated by butyrate producers early in life lowers the risk of developing allergies by promoting the maturation and proper function of the GB.

Importantly, several articles included in this Research Topic provided important evidence on key factors that regulate GB function such as the commensal microbiota composition, psychological stress, diet and other environmental factors (i.e., cigarette smoking). Two reviews summarized the importance of the gut microbiota in regulating GB function. For example, Wang et al. highlighted the importance of dysbiosis in shaping intestinal epithelial cells and mucosal immunity, thereby reducing their ability to contain microbial pathogens and prevent gut-derived sepsis. Antonini et al. discussed how dysbiosis may lead to extra-intestinal autoimmune diseases by altering GB integrity and favoring the passage of bacterial components that could reach peripheral organs to activate autoimmune responses in the central nervous system. Not only bacterial dysbiosis but also alteration of the fungal component can lead to GB damage, inflammation and T1D as shown by Honkannen et al. The review by Taleb et al. reported evidence that dietary components such as tryptophan by regulating the activity of the enzyme indoleamine 2,3 dioxygenase 1 (IDO1) impacts gut immunity and also the GB defense mechanism via the induction of antimicrobial peptides, mucins and tight junction protein expression. In light of the recent evidence on the importance of the brain-gut axis and the ability of the CNS to regulate GB function, Ilchmann-Diounou et al. reviewed published work on how psychological stress promotes intestinal inflammation and breaks the integrity of the intestinal epithelial and mucosal barriers. Finally, Berkowitz et al. demonstrated that the administration of cigarette smoke condensate compromises the gut barrier architecture by inducing Paneth cell damage and reducing the GB defense system. The review by Klepsch et al. dissected the possible mechanism by which these diverse external factors regulate GB integrity. Specifically, the Authors discussed the complex biology and immunoregulatory function of the nuclear receptor (NR) family, a class of receptors that are highly expressed in the gastrointestinal tract and function as sensors of microbial metabolites as well as nutrients and food components. Several NRs have been found capable of regulating gut immune homeostasis by sustaining the integrity of the physical and biological gut barriers.

Restoration of GB integrity could be an innovative therapeutic approach to treat different immune-mediated diseases such as autoimmune diseases and allergies. In this Research Topic an innovative therapeutic approach in this direction was suggested by Xie et al. The Authors showed that administration of porcine β-defensin 129, an anti-microbial peptide normally present in the mucus layer, reinforces GB integrity and reduces intestinal inflammation.

## Author contributions

MF: Writing – review & editing, Writing – original draft. VP: Writing – review & editing, Writing – original draft.
